# Cerebrospinal Fluid and Arterial Acid–Base Equilibrium of Spontaneously Breathing Patients with Aneurismal Subarachnoid Hemorrhage

**DOI:** 10.1007/s12028-022-01450-1

**Published:** 2022-02-23

**Authors:** Thomas Langer, Francesco Zadek, Marco Carbonara, Alessio Caccioppola, Serena Brusatori, Tommaso Zoerle, Francesco Bottazzini, Chiara Ferraris Fusarini, Adriana di Modugno, Alberto Zanella, Elisa R. Zanier, Roberto Fumagalli, Antonio Pesenti, Nino Stocchetti

**Affiliations:** 1 Department of Medicine and Surgery, University of Milan-Bicocca, Monza, Italy; 2 Department of Anesthesia and Intensive Care Medicine, Niguarda Ca’ Granda, Milan, Italy; 3 Department of Pathophysiology and Transplantation, University of Milan, Milan, Italy; 4 Department of Anesthesia, Critical Care and Emergency, Fondazione IRCCS Ca’ Granda Ospedale Maggiore Policlinico, Milan, Italy; 5 Central Laboratory, Fondazione IRCCS Ca’ Granda Ospedale Maggiore Policlinico, Milan, Italy; 6 Laboratory of Acute Brain Injury and Therapeutic Strategies, Department of Neuroscience, Istituto Di Ricerche Farmacologiche Mario Negri IRCCS, Milan, Italy

**Keywords:** Acid–base equilibrium, Cerebrospinal fluid, Blood, Subarachnoid hemorrhage, Electrolyte disturbances

## Abstract

**Background:**

Hyperventilation resulting in hypocapnic alkalosis (HA) is frequently encountered in spontaneously breathing patients with acute cerebrovascular conditions. The underlying mechanisms of this respiratory response have not been fully elucidated. The present study describes, applying the physical–chemical approach, the acid-base characteristics of cerebrospinal fluid (CSF) and arterial plasma of spontaneously breathing patients with aneurismal subarachnoid hemorrhage (SAH) and compares these results with those of control patients. Moreover, it investigates the pathophysiologic mechanisms leading to HA in SAH.

**Methods:**

Patients with SAH admitted to the neurological intensive care unit and patients (American Society of Anesthesiologists physical status of 1 and 2) undergoing elective surgery under spinal anesthesia were enrolled. CSF and arterial samples were collected simultaneously. Electrolytes, strong ion difference (SID), partial pressure of carbon dioxide (PCO_2_), weak noncarbonic acids (A_TOT_), and pH were measured in CSF and arterial blood samples.

**Results:**

Twenty spontaneously breathing patients with SAH and 25 controls were enrolled. The CSF of patients with SAH, as compared with controls, was characterized by a lower SID (23.1 ± 2.3 vs. 26.5 ± 1.4 mmol/L, *p* < 0.001) and PCO_2_ (40 ± 4 vs. 46 ± 3 mm Hg, *p* < 0.001), whereas no differences in A_TOT_ (1.2 ± 0.5 vs. 1.2 ± 0.2 mmol/L, *p* = 0.95) and pH (7.34 ± 0.06 vs. 7.35 ± 0.02, *p* = 0.69) were observed. The reduced CSF SID was mainly caused by a higher lactate concentration (3.3 ± 1.3 vs. 1.4 ± 0.2 mmol/L, *p* < 0.001). A linear association (*r* = 0.71, *p* < 0.001) was found between CSF SID and arterial PCO_2_. A higher proportion of patients with SAH were characterized by arterial HA, as compared with controls (40 vs*.* 4%, *p* = 0.003). A reduced CSF-to-plasma difference in PCO_2_ was observed in nonhyperventilating patients with SAH (0.4 ± 3.8 vs*.* 7.8 ± 3.7 mm Hg, *p* < 0.001).

**Conclusions:**

Patients with SAH have a reduction of CSF SID due to an increased lactate concentration. The resulting localized acidifying effect is compensated by CSF hypocapnia, yielding normal CSF pH values and resulting in a higher incidence of arterial HA.

**Supplementary Information:**

The online version contains supplementary material available at 10.1007/s12028-022-01450-1.

## Introduction

According to Stewart’s physical–chemical approach [1], the acid–base equilibrium of biological fluids is regulated independently by three variables: the partial pressure of carbon dioxide (PCO_2_), the strong ion difference (SID), and the total amount of weak nonvolatile buffers (A_TOT_). Cerebrospinal fluid (CSF) in normal conditions is a clear fluid surrounding the brain, characterized by a very low concentration of proteins. It is produced mainly by the choroid plexus and reabsorbed by the arachnoid villi [2]. Given its negligible amount of proteins, only two variables regulate the acid–base equilibrium of CSF, namely PCO_2_ and SID [3]. The acid–base equilibrium of this small and very particular extracellular fluid compartment, i.e., CSF, is of particular importance because it contributes significantly to the regulation of cerebral blood flow [4] and spontaneous breathing activity [5].

Hyperventilation resulting in hypocapnic alkalosis (HA) is frequently encountered in spontaneously breathing patients with acute cerebrovascular conditions [6–8] or severe brain injuries [9]. The pathophysiologic mechanisms underlying this abnormal respiratory response have not been fully elucidated. On one hand, the existence of a neurogenic stimulus causing a pathologic activation of the respiratory centers has been hypothesized [8, 10, 11]. On the other hand, the mixing of CSF with exogenous blood could determine an acidification of the extracellular cerebral milieu, fostering an increase in minute ventilation to restore normal pH values in proximity of the respiratory centers [7].

The aim of the present study was to (1) describe, applying Stewart’s physical–chemical approach, the acid–base characteristics of CSF and arterial plasma of spontaneously breathing patients with subarachnoid hemorrhage (SAH); (2) compare these results with those of healthy controls; and (3) investigate the pathophysiologic mechanisms leading to HA in SAH. We hypothesized that patients with SAH would be characterized by a local CSF metabolic acidosis (lower CSF SID), which would favor the development of hypocapnia.

## Methods

### Institutional Review Board and Patient Consent

The study protocol and the informed consent process were approved by the Ethical Committee Milano Area B (protocol number: 25_2017bis), and written informed or deferred consent was obtained from patients with SAH and controls.

### Patients with Spontaneous SAH

Patients with SAH admitted to the neurological intensive care unit (ICU) of our hospital, in whom an external ventricular drain was placed for clinical reasons, with preserved spontaneous respiratory activity were enrolled. Details about our clinical practice regarding SAH treatment can be found elsewhere [12]. Both nonintubated patients and intubated patients on pressure support ventilation were studied. Age younger than 18 years and pregnancy constituted exclusion criteria. In each patient, CSF and arterial blood samples were collected simultaneously as close as possible to the day of SAH onset. A CSF sample of 1.5 mL was drawn anaerobically and under strict asepsis from the external ventricular drain after the dead space of the catheter was discarded. Simultaneously, an arterial blood sample was collected from an indwelling arterial catheter. In addition, the following clinical data were recorded: medical history, the severity of bleeding (modified Fisher scale), day from bleeding, neurological assessment at ICU admission (World Federation Neurological Surgeons Scale), and Glasgow Coma Scale score on the study day [13].

### Control Population

Patients undergoing spinal anesthesia for elective surgery constituted the control group. Exclusion criteria were American Society of Anesthesia (ASA) physical status above 2, age younger than 18, pregnancy, and known neurological, renal, or respiratory disease. Before the local anesthetic injection, 1.5 mL of CSF was collected anaerobically from the needle used for spinal anesthesia. The radial artery was punctured to collect an arterial blood sample. Anesthesiologists were asked to limit intravenous fluids prior to spinal anesthesia and to choose balanced solutions to avoid intravenous-fluids-related acid–base derangements [14, 15].

### Analysis of CSF and Blood

Acid–base and electrolytes of CSF and arterial blood samples were immediately measured (ABL800 FLEX, Radiometer, Copenhagen, Denmark). Magnesium, phosphate, and albumin levels (COBAS 8000, Roche Diagnostics GmbH, Mannheim, Germany); hemoglobin concentration; and red and white blood cell counts (XN-9000 V, Sysmex Corporation, Kobe, Japan) were analyzed for CSF and blood samples. Plasma and CSF osmolarity were measured (Osmometer MIR 300-P, E. Mires, Milan, Italy).

For each CSF and blood sample, the SID was calculated as indicated in Eq. 1 [16]:

1 $$SID = \left[ {Na^{ + } \left] { + } \right[K^{ + } \left] { + 2 \times } \right[Ca^{2 + } \left] { + 2 \times } \right[Mg^{2 + } \left] { - } \right[Cl^{ - } \left] { - } \right[Lac^{ - } } \right],$$

where Na^+^, K^+^, Ca^2+^, Cl^−^, and Lac^−^ refer to plasma sodium, potassium, ionized calcium, chloride, and lactate concentrations expressed as millimolar.

Moreover, the dissociated part of noncarbonic weak acid was computed using Eq. 2 [16]:

2 $$\left[ {A^{ - } } \right] = \left[ {Alb} \right] \times \;\left( {pH \times \;0.1204 - 0.625} \right) + \left[ {Pi} \right] \times \;\left( {pH \times \;0.309 - 0.469} \right),$$

where [A^−^] is expressed in mmol/L, [Alb] is the plasma concentration of albumin expressed in g/L, [P*i*] is the plasma concentration of phosphates expressed in mmol/L, and pH denotes arterial or cerebrospinal pH.

The total amount of weak acids [A_TOT_] was calculated, rearranging the dissociation equation as reported in Eq. 3:

3 $$[A_{TOT} ] = [A^{ - } ] \times \;\frac{{1 + 10^{{(pH - pK_{A} )}} }}{{10^{{(pH - pK_{A} )}} }},$$

where pH is the arterial or cerebrospinal pH, and pK_A_ is equal to 6.8 and represents the negative logarithm to base 10 of the dissociation constant of noncarbonic buffers.

The difference between CSF and plasma SID and CSF and plasma PCO_2_ was computed and termed ΔSID and ΔPCO_2_, respectively.

4 $$\Delta SID \left[ {mmol/L} \right] = \left[ {SID} \right]_{CSF } - \left[ {SID} \right]_{Pl}$$

5 $${\Delta }PCO_{2} \left[ {mm\ Hg} \right] = PCO_{2\, CSF} - PCO_{2\, Pl}$$

The ratios between CSF and arterial plasma were calculated as previously described [11].

6 $$Ratio = [\ ]_{CSF} /[\ ]_{Pl}$$

Bicarbonate ion concentration ([HCO_3_^−^]) was calculated from pH and PCO_2_ according to the Henderson–Hasselbalch equation ($pH\; = \;pK\; + \;\log_{10} \frac{{[HCO_{3}^{ - } ]}}{{\alpha \ \times \;PCO_{2} }}$), assuming pK = 6.1 and α = 0.0306 for plasma and pK = 6.13 and α = 0.0318 for CSF [17].

7 $$HCO_{3}^{ - } = \alpha \times PCO_{2} \times 10^{{\left( {pH {-} pK} \right)}}$$

### Clinical Definition of HA

Patients with an arterial PCO_2_ < 35 mm Hg and an arterial pH > 7.45 were included in the HA subgroup, whereas patients who did not present these features were included in the subgroup without HA.

### Clinical Outcomes

The Glasgow Outcome Scale Extended (GOS-E) [18, 19] was scored at 12 months. A good clinical outcome was defined as a GOS-E score ≥ 6. Information regarding clinical outcome was gathered over the phone, directly, or through the caregiver. Data about the incidence of clinical vasospasm, defined as new neurological symptoms associated with confirmed vasospasm diagnosed by a neuroradiologist [20], were collected from the patients’ medical charts.

### Sample Size and Statistical Analysis

Data analysis and statistical plans were written and filed with the institutional review board before data were accessed. We calculated that a sample of 40 patients (20 patients per group) would provide a statistical power of 0.9 with an α error of 0.05 to detect a difference in SID of 3 mEq/L, assuming a standard deviation of 2.5 mEq/L. Given the novelty of the measurement of some CSF variables, we decided to increase the number of control patients to 25.

Comparison between continuous variables was performed via Student’s *t*-test or the Mann–Whitney rank-sum test, as appropriate. Differences between categorical variables were assessed using the χ^2^ test. The relationship between quantitative or categorical variables was investigated using linear and multilinear regression, as appropriate. Data are expressed as mean ± standard deviation or median and interquartile range, unless otherwise stated. Statistical significance was defined as *p* < 0.05. Analysis was performed with SAS v.9.2 (SAS Institute, Inc., Cary, NC) and SigmaPlot v.12.0 (Systat Software, San Jose, CA). The Strengthening the Reporting of Observational Studies in Epidemiology checklist was used.

## Results

### Description of the Study Population

Patients were enrolled between February 2017 and October 2018. Twenty spontaneously breathing patients with aneurismal SAH (aged 57 ± 11 years, 16 women, body mass index 25 [23–29]) were enrolled and studied on day 2 (2–4) after bleeding. The median World Federation Neurological Surgeons Scale score at ICU admission was 3.5 (1–4), the median modified Fisher scale score was 4 (3–4), and the median Glasgow Coma Scale core on the study day was 12 (9–15). Ten patients were not intubated and were spontaneously breathing. The remaining ten were intubated on pressure support ventilation. At the sampling time, no patient exhibited neurogenic breathing patterns [21] or hypoxic respiratory failure (PaO_2_/FiO_2_ = 352 ± 121). Twenty-five healthy controls were enrolled (aged 56 ± 16 years, 9 women, body mass index 24 [23–25], 8 patients with an ASA physical status of 1, 17 patients with an ASA physical status of 2). The only statistically significant difference found between controls and patients with SAH was a higher prevalence of female sex in patients with SAH (80% vs. 36%, *p* = 0.004). Comorbidities of patients with SAH and controls are reported in Table S1.

### Description of Cerebrospinal and Arterial Acid–Base Characteristics

Cerebrospinal and arterial acid–base findings are summarized in Tables 1 and 2. CSF of patients with SAH was characterized by an increased presence of red and white blood cells, whereas no red blood cells and only 2 ± 2 white blood cells per microliter were found in controls. The CSF of patients with SAH, as compared with controls, had lower SID and PCO_2_ and similar A_TOT_. No difference in CSF pH was observed. The reduced CSF SID was caused mainly by an increased lactate concentration (*p* < 0.001). On the plasma side (Table 2), similar SID and PCO_2_ values were recorded. The plasma of patients with SAH was characterized by lower A_TOT_, as compared with controls, leading to a significantly higher arterial pH. The reduction in A_TOT_ observed in patients with SAH was caused both by lower albumin (4.2 ± 0.4 vs*.* 3.5 ± 0.4 g/dL, *p* < 0.001) and phosphate (3.2 ± 0.6 vs*.* 2.5 ± 0.8 mg/dL, *p* = 0.003) concentrations.

**Table 1:** Acid–base characteristics of cerebrospinal fluid of the study population

Variables	Controls (*n* = 25)	SAH (*n* = 20)	*p* value
pH	7.35 ± 0.02	7.34 ± 0.06	0.69
PCO_2_ (mm Hg)	46 ± 3	40 ± 4	< 0.001
A_TOT_ (mmol/L)	1.2 ± 0.2	1.2 ± 0.5	0.95
SID (mmol/L)	26.5 ± 1.4	23.1 ± 2.3	< 0.001
Na^+^ (mmol/L)	141 ± 1	143 ± 3	0.007
K^+^ (mmol/L)	2.8 ± 0.1	2.6 ± 0.5	0.07
Ca^2+^ (mmol/L)	0.99 ± 0.02	0.98 ± 0.06	0.22
Mg^2+^ (mmol/L)	1.10 ± 0.04	1.15 ± 0.06	0.002
Cl^−^ (mmol/L)	120 ± 2	123 ± 4	0.001
Lac^−^ (mmol/L)	1.4 ± 0.2	3.3 ± 1.3	< 0.001
HCO_3_^−^ (mmol/L)	24.7 ± 1.5	21.7 ± 2.4	< 0.001
Phosph^−^ (mEq/L)	0.77 ± 0.08	0.66 ± 0.20	0.02
Alb^−^ (mEq/L)	0.19 ± 0.14	0.30 ± 0.23	0.05
Osmolarity (mOsm/L)	285 ± 3	290 ± 7	0.001
Gluc (mg/dL)	59 ± 7	74 ± 14	< 0.001
Hb (g/dL)	0.0 ± 0.0	0.3 ± 0.3	< 0.001
RBC (× 10^6^/µL)	0.00 ± 0.00	0.11 ± 0.12	< 0.001
WBC (cells/µL)	2 ± 2	359 ± 562	0.003

Data are expressed as mean ± standard deviation

Alb^−^, ionized albumin concentration, A_TOT_, total amount of weak noncarbonic acids, Ca^2+^, ionized calcium concentration, Cl^−^, chloride concentration, Gluc, glucose concentration, Hb, hemoglobin concentration, HCO_3_^−^, bicarbonate concentration, K^+^, potassium concentration, Lac^−^, lactate concentration, Mg^2+^, magnesium concentration, Na^+^, sodium concentration, PCO_2_, partial pressure of carbon dioxide, Phosph^−^, ionized phosphate concentration, RBC, red blood cell count, SAH, aneurismal subarachnoid hemorrhage, SID, strong ion difference, WBC, white blood cell count

**Table 2:** Acid–base characteristics of arterial plasma of the study population

Variables	Controls (*n* = 25)	SAH (*n* = 20)	*p* value
pH	7.42 ± 0.02	7.45 ± 0.04	< 0.001
PCO_2_ (mm Hg)	39 ± 3	37 ± 6	0.15
A_TOT_ (mmol/L)	16.3 ± 1.5	13.5 ± 1.6	< 0.001
SID (mmol/L)	36.2 ± 1.8	37.2 ± 3.7	0.23
PO_2_ (mm Hg)	99 ± 49	111 ± 28	0.35
Na^+^ (mmol/L)	138 ± 2	142 ± 4	< 0.001
K^+^ (mmol/L)	4.0 ± 0.3	3.5 ± 0.2	< 0.001
Ca^2+^ (mmol/L)	1.22 ± 0.03	1.17 ± 0.04	< 0.001
Mg^2+^ (mmol/L)	0.84 ± 0.07	0.85 ± 0.07	0.88
Cl^−^ (mmol/L)	109 ± 2	111 ± 5	0.03
Lac^−^ (mmol/L)	1.1 ± 0.4	1.0 ± 0.5	0.25
HCO_3_^−^ (mmol/L)	25.0 ± 2.0	25.5 ± 2.9	0.19
Phosph^−^ (mEq/L)	1.9 ± 0.3	1.5 ± 0.5	0.004
Alb^−^ (mEq/L)	11.3 ± 1.1	9.6 ± 1.1	< 0.001
SBE (mmol/L)	0.5 ± 2.0	1.6 ± 2.6	0.09
Osmolarity (mOsm/L)	281 ± 3	291 ± 8	< 0.001
Gluc (mg/dL)	100 ± 11	129 ± 20	< 0.001
Gluc ratio (%)	59 ± 6	58 ± 8	0.55
Hb (g/dL)	14.0 ± 1.6	11.4 ± 1.6	< 0.001
RBC (× 10^6^/µL)	4.5 ± 0.5	3.6 ± 0.7	< 0.001
WBC (× 10^3^/µL)	5.9 ± 1.0	10.2 ± 2.4	< 0.001

Data are expressed as mean ± standard deviation

Alb^−^, ionized albumin concentration, A_TOT_, total amount of weak noncarbonic acids, Ca^2+^, ionized calcium concentration, Cl^−^, chloride concentration, Gluc, glucose concentration, Hb, hemoglobin concentration, HCO_3_^−^, bicarbonate concentration, K^+^, potassium concentration, Lac^−^, lactate concentration, Mg^2+^, magnesium concentration, Na^+^, sodium concentration, PCO_2_, partial pressure of carbon dioxide, Phosph^−^, ionized phosphate concentration, PO_2_, partial pressure of oxygen, RBC, red blood cell count, SAH, aneurismal subarachnoid hemorrhage, SBE, standard base excess, SID, strong ion difference, WBC, white blood cell count

**Fig. 1:**
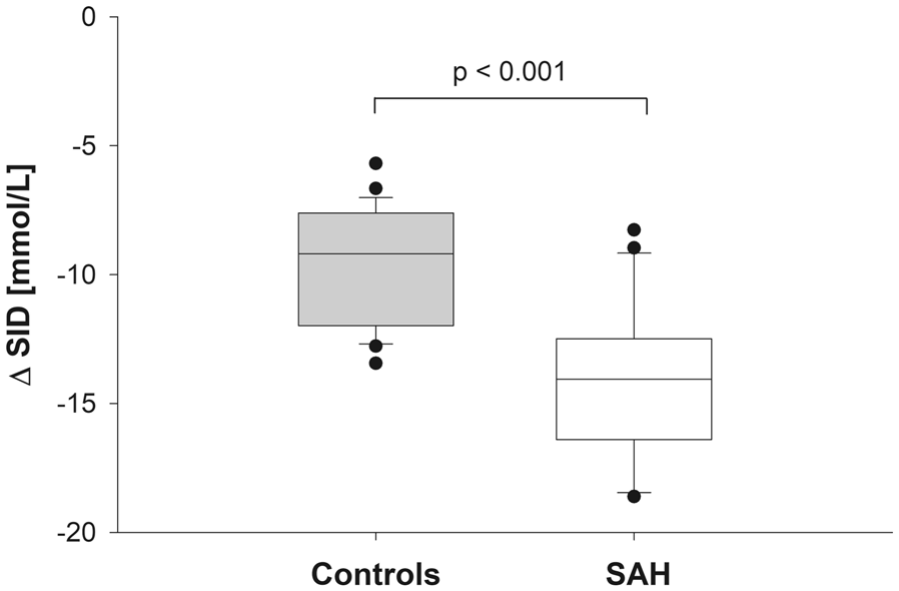
ΔSID in controls and patients with SAH. Box-plot graph representing the CSF-to-plasma SID difference (ΔSID). Whiskers represent 10th and 90th percentiles. CSF cerebrospinal fluid, SAH aneurismal subarachnoid hemorrhage, SID strong ion difference

The ΔSID, i.e., the difference between CSF and plasma SID, differed significantly between the two groups (Fig. 1). Furthermore, ΔPCO_2_, the CSF-to-plasma difference in PCO_2_, showed significantly different values between controls and patients with SAH (7.5 ± 2.5 vs. 3.4 ± 5.2 mm Hg, *p* = 0.001). Measured osmolarity of both plasma (*p* < 0.001) and CSF (*p* = 0.001) was significantly higher in patients with SAH.

### HA and Underlying Mechanisms

The incidence of HA was higher in spontaneously breathing patients with SAH, as compared with controls (40% vs. 4%, *p* = 0.003).

When comparing hypocapnic and nonhypocapnic patients with SAH, no differences in demographic and clinical variables were observed (Table S2). Arterial and CSF acid–base characteristics of patients with HA and those without HA are summarized in Table S3 and Table 3, respectively.

**Table 3:** Acid–base characteristics of cerebrospinal fluid of the SAH population

Variables	Nonhypocapnic alkalosis (*n* = 12)	Hypocapnic alkalosis (*n* = 8)	*p* value
pH	7.36 ± 0.05	7.33 ± 0.06	0.26
PCO_2_ (mm Hg)	41 ± 4	39 ± 4	0.47
A_TOT_ (mmol/L)	1.3 ± 0.5	1.1 ± 0.3	0.18
SID (mmol/L)	24.2 ± 1.7	21.3 ± 1.9	0.002
Na^+^ (mmol/L)	143 ± 5	143 ± 1	0.98
K^+^ (mmol/L)	2.5 ± 0.4	2.6 ± 0.7	0.78
Ca^2+^ (mmol/L)	1.00 ± 0.04	0.96 ± 0.07	0.11
Mg^2+^ (mmol/L)	1.17 ± 0.07	1.13 ± 0.04	0.15
Cl^−^ (mmol/L)	122 ± 5	125 ± 3	0.13
Lac^−^ (mmol/L)	3.4 ± 1.4	3.1 ± 1.2	0.60
HCO_3_^−^ (mmol/L)	22.7 ± 2.1	20.4 ± 2.3	0.03
Phosph^−^ (mEq/L)	0.68 ± 0.23	0.62 ± 0.17	0.56
Alb^−^ (mEq/L)	0.37 ± 0.26	0.19 ± 0.12	0.09
Osmolarity (mOsm/L)	290 ± 9	290 ± 2	0.95
Gluc (mg/dL)	73 ± 15	76 ± 14	0.72
Hb (g/dL)	0.4 ± 0.4	0.3 ± 0.3	0.70
RBC (× 10^6^/µL)	0.12 ± 0.14	0.09 ± 0.07	0.59
WBC (cells/µL)	326 ± 572	408 ± 588	0.76
ΔSID (mmol/L)	14.4 ± 3.7	13.8 ± 1.2	0.65
ΔPCO_2_ (mm Hg)	0.4 ± 3.8	7.8 ± 3.7	< 0.001

Data are expressed as mean ± standard deviation

Alb^−^, ionized albumin concentration, A_TOT_, total amount of weak noncarbonic acids, Ca^2+^, ionized calcium concentration, Cl^−^, chloride concentration, Gluc, glucose concentration, Hb, hemoglobin concentration, HCO_3_^−^, bicarbonate concentration, K^+^, potassium concentration, Mg^2+^, magnesium concentration, Na^+^, sodium concentration, PCO_2_, partial pressure of carbon dioxide, Phosph^−^, ionized phosphate concentration, RBC, red blood cell count, SAH, aneurismal subarachnoid hemorrhage, SID, strong ion difference, WBC, white blood cell count

Although CSF SID was lower (*p* = 0.002) in patients with HA, CSF PCO_2_ and pH did not differ significantly between groups. Notably, CSF electrolytes did not differ significantly between patients with HA and those without HA. Regarding plasma, in addition to the differences in PCO_2_ and pH due to the selection criteria, arterial SID was significantly lower in patients with HA.

A lower ΔPCO_2_ was measured in patients without HA compared with those with HA (Table 3). Of note, the ΔPCO_2_ of patients with HA was similar to values measured in the control group (*p* = 0.78).

### The Interplay Between Variables and pH

Linear and multilinear regression analyses were used to investigate the association between variables both in controls and in patients with SAH. In controls, no association was found at univariate analysis between CSF SID (*p* = 0.93) or CSF PCO_2_ (*p* = 0.13) and CSF pH.

**Fig. 2:**
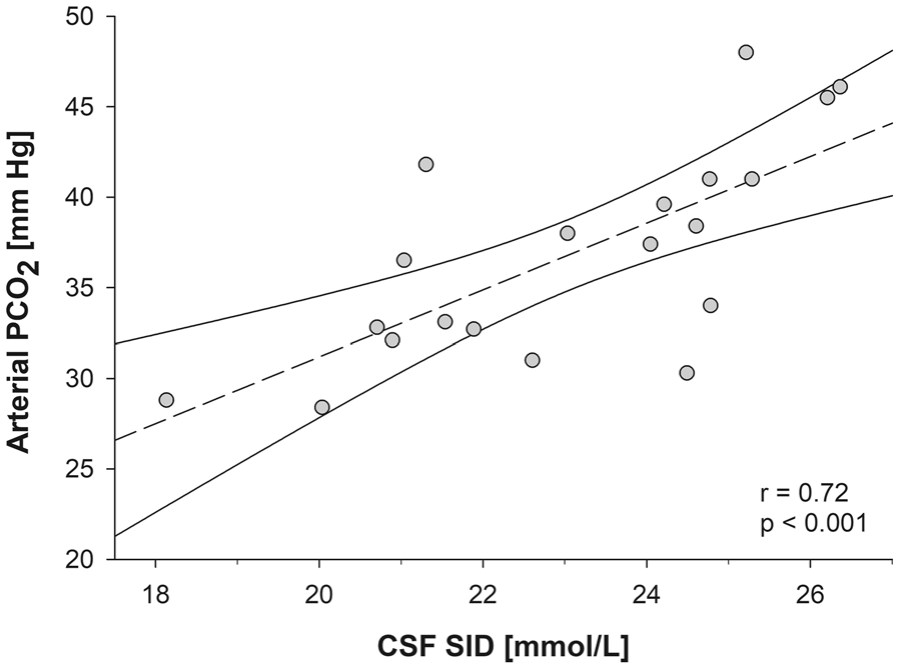
Scatter plot graph of CSF SID and arterial PCO_2_ in patients with SAH. Scatter Plot representing the association between CSF SID and arterial PCO_2_ in patients with SAH. Linear regression model (dashed line) is represented with 95% confidence interval. Equation of the linear model: Arterial PCO_2_ = 1.84 × CSF SID − 5.7. CSF cerebrospinal fluid, PCO_2_ partial pressure of carbon dioxide, SAH aneurismal subarachnoid hemorrhage, SID strong ion difference

In patients with SAH, both CSF SID and PCO_2_ showed a significant association with CSF pH (*r* = 0.54 [*p* = 0.01] and *r* =  − 0.59 [*p* = 0.006], respectively). Both variables remained independently associated at the multilinear regression analysis (*r*^2^ = 0.79, *p* < 0.001; Table S4). At univariate analysis, CSF sodium (*r* =  − 0.55, *p* = 0.01), chloride (*r* =  − 0.62, *p* = 0.003), and lactate (*r* =  − 0.60, *p* = 0.005) were significantly associated with CSF pH. However, at multilinear regression analysis, only lactate was independently associated with CSF pH (Table S5). Lastly, in the overall SAH population, a positive correlation between CSF SID and arterial PCO_2_ was found (Fig. 2). Similarly, a negative correlation between CSF SID and arterial pH was observed (*r* =  − 0.62, *p* < 0.004; Fig. S1).

### Clinical Outcome

Six patients with SAH experienced an episode of clinical vasospasm; two of these patients died in the ICU, and four patients were lost at follow-up. Plasma and CSF acid–base characteristics of patients who developed vasospasm did not differ significantly from those of patients who did not (Tables S6 and S7). No significant association was observed between CSF SID (or ΔSID) and the modified Fisher scale score by Pearson’s correlation (data not shown). The median 12-month GOS-E score was 6 (3–8). No difference (*p* = 0.64) was observed in GOS-E scores between patients with HA and those without HA. A good clinical outcome (GOS-E score ≥ 6) was observed in 10 of 16 patients. Patients with worse clinical outcomes had higher CSF lactate concentrations (4.5 ± 1.4 vs. 2.7 ± 0.9, *p* = 0.006).

## Discussion

In the present study, we used the physicochemical approach to acid–base [1] to describe the acid–base characteristics of both arterial blood and CSF of spontaneously breathing patients with SAH and compared these results with those of a control population, i.e., healthy, spontaneously breathing patients undergoing elective surgery under spinal anesthesia. The main finding of our study was that patients with SAH had lower CSF SID, mainly due to an increased concentration of lactate. Arterial SID did not differ between the two populations. Therefore, when blood SID was subtracted from CSF SID (ΔSID), significantly different values were observed in patients with SAH and controls (Fig. 1). This finding clearly points toward a primary metabolic acid–base disorder caused by intracranial bleeding and compartmentalized in the cerebral/CSF environment. Despite the acidifying effect of a reduced CSF SID, CSF pH was similar in the two populations, as CSF PCO_2_ was significantly reduced in patients with SAH. The respiratory compensation of this local metabolic acidosis led to blood HA in 40% of the SAH population.

Isolated CSF acidosis has been induced in several animal models [22–25] by directly injecting acids into the ventricular system. In these studies, local CSF acidification triggered the respiratory chemoreceptors and caused an increase in minute ventilation to restore CSF pH through a reduction in CSF PCO_2_ [22]. Given the anatomical and functional separation between blood and CSF, the acids injected locally did not reach the bloodstream, which was therefore unaffected by the acid–base disorder. Consequently, the compensatory hyperventilation corrected CSF pH and led to a systemic HA of the blood.

Although the presence of HA frequently characterizes patients with cerebrovascular conditions, only a few reports [5, 11, 26] investigated the underlying mechanisms. We hypothesized that a local CSF metabolic acidosis, i.e., a reduction in CSF SID, could partially explain the finding. We therefore studied the blood/CSF electrolytes and acid–base of healthy patients undergoing spinal anesthesia and considered these data, for the present study, as baseline values, i.e., normal reference values before the SAH.

Our results from the control population regarding CSF electrolytes and acid–base variables are in line with the available literature [11, 27–29]: normal CSF has a pH around 7.35 with a SID close to 26 mmol/L. In CSF, the protein concentration is negligible, and electrical neutrality is guaranteed by a higher chloride concentration as compared with plasma (120 vs. 109 mmol/L).

The CSF acid–base characteristics of patients with SAH differed significantly as compared with controls. In particular, in patients with SAH, we observed a lower CSF SID, which favors acidification. The causes of the reduced SID are likely multifactorial. First, spontaneous SAH causes the sudden release of blood in the subarachnoid space, frequently including the cerebral ventricles. Although blood has a higher pH and SID, as compared with CSF (Tables 1 and 2), the presence of living red blood cells reaching the CSF could increase CSF lactate concentration because of their anaerobic metabolism [7]. Second, cerebrovascular events frequently lead to cerebral hypoperfusion/ischemia fostering the release of metabolites (mainly lactate) from suffering cerebral tissue [30–33]. Finally, therapies administered in the critical care setting might affect the electrolyte composition of the extracellular fluid, including CSF. Indeed, although the increase in CSF lactate concentration observed in our patients was a consequence of SAH, the alterations of other electrolytes, such as sodium and chloride, could be secondary to the use of isotonic/hypertonic NaCl or other crystalloids. Despite the difference in CSF SID, CSF pH did not differ between the two populations because CSF PCO_2_ was lower in patients with SAH. This finding might be interpreted as a compensatory hyperventilation triggered by the local CSF acidification.

Similar considerations can be made when analyzing the differences in arterial blood composition (Table 2). Indeed, some differences are likely a consequence of medical treatment. For instance, we observed higher concentrations of sodium and chloride and lower concentrations of albumin, hemoglobin, and phosphates. All these findings can be attributed (at least in part) to intravenous fluids with slightly hypertonic, chloride-rich fluids. In this population, supranormal sodium concentrations and plasma osmolarity are therapeutic targets to reduce the cerebral swelling risk [34]. Overall, the plasma SID of patients with SAH was similar to that of controls. On the other hand, patients with SAH had lower concentrations of weak noncarbonic acids, mainly due to hypoalbuminemia. According to Stewart’s approach, a decrease in weak noncarbonic acids unbalances the acid–base equilibrium toward alkalosis. Of note, hypoalbuminemia is very frequent in critically ill patients [35], in particular in patients with neurologic disorders, given the relative contraindication to its exogenous supplementation [36, 37].

Patients were divided according to the presence/absence of HA (Table 3). This condition was frequent (40%) in patients with SAH, whereas it was present only in one control patient. Per definition, patients with HA had lower arterial PCO_2_ and higher arterial pH. Moreover, patients with HA had a lower arterial SID, mainly due to a higher chloride concentration. It is conceivable that the renal compensation of HA might have contributed to this finding [38].

On the CSF side, patients with HA had significantly lower CSF SID as compared with nonhypocapnic patients. The reduced SID was not attributable to a single major electrolyte derangement: several nonsignificant electrolyte differences were observed.

Another striking difference between patients with HA and those without HA was ΔPCO_2_, i.e., the CSF-to-plasma difference in PCO_2_. In patients with HA, ΔPCO_2_ had normal values, i.e., values similar to those of controls. On the contrary, ΔPCO_2_ was significantly reduced in patients without HA (Table 3). Sambrook et al. [11] already described a reduced ΔPCO_2_ in a case series of patients with SAH, and a reduction of cerebral metabolism was hypothesized to explain this finding [32, 39]. A possible interpretation is the following: a lower cerebral metabolism could characterize patients without HA, leading to a lower local carbon dioxide production and thus a lower difference in PCO_2_ as compared with arterial blood. Consequently, in patients without HA, CSF pH is close to normal as a result of a lower CSF SID and lower CSF PCO_2_. Apparently, however, the lower CSF PCO_2_ is not the result of hyperventilation, because arterial PCO_2_ is normal, but could be the expression of a reduced local carbon dioxide production. Of note, using positron emission tomography, Carpenter and colleagues [39] documented a reduced cerebral metabolism in patients with SAH.

We used linear and multilinear regression analyses to investigate the association between acid–base variables in the two extracellular compartments. Interestingly, no linear association was found in controls between both CSF PCO_2_ and SID and CSF pH, suggesting the presence of a stable physiologic equilibrium. On the contrary, in patients with SAH, there was an association between both CSF SID and PCO_2_ and CSF pH at linear and multilinear regression. Interestingly, only lactate was independently associated with CSF pH at multilinear regression. In the overall SAH population, CSF SID was linearly correlated with both arterial PCO_2_ (Fig. 2) and pH, suggesting its key role in determining the CSF acid–base disorder and thus favoring the respiratory compensation.

### Clinical Implications and Future steps

The presence of hypocapnia in patients with SAH has been hypothesized to be a marker of severity and to be associated with worse outcomes [40]. The idea is that a primary hyperventilation might lead to cerebral hypocapnia and alkalosis, leading to a reduced cerebral blood flow and possibly fostering the development of vasospasm. We did not find any difference regarding clinical outcomes between patients with HA and those without HA. Similarly, we did not find an association between CSF SID and the modified Fisher scale score. This finding might be due to both the limited sample size and the fact that the modified Fisher scale score is based on a computed tomography scan obtained on the day of intensive care admission, whereas the samples are obtained a median of 2 days after bleeding, after the placement of an external ventricular drain.

Our data suggest that the response to a local CSF acidification might differ among patients with SAH and that cerebral metabolism, and therefore local CO_2_ production, might be an important variable. An additional clinical message of our study could be the following: patients with SAH with an external ventricular drain have an easy and safe access to CSF. This particular extracellular fluid (CSF) contains several physiologic information linked to spontaneous breathing activity and cerebral blood flow. In a patient population characterized by an acute neurologic condition, this fluid might even gain more importance, including, possibly, a prognostic role. Our limited sample size is of course insufficient to draw inferences about the prognostic role of a single value or of its time course. However, we think that it might be a good research question for future studies.

### Limitations

We need to acknowledge some limitations of our study. First, a higher presence of men characterized our controls, as compared with the SAH population. Although hormones certainly play a role in respiratory regulation, this is more evident during pregnancy, which was an exclusion criterion for our study. Second, we sampled CSF in different sites, i.e., lumbar in controls and ventricular in patients with SAH. Differences in PCO_2_ and pH of the two sites are minimal in physiologic conditions [41]. Therefore, it is conceivable to consider the lumbar CSF samples of controls to be similar to their ventricular CSF.

On the contrary, meaningful differences between lumbar and ventricular CSF could be present in patients with SAH [29, 42, 43]. For this reason, we analyzed only ventricular CSF in patients with SAH because this is the extracellular fluid in close contact with the chemosensitive area [44–46]. Moreover, we did not collect and report information regarding the type and amount of intravenous fluids administered to patients with SAH. As discussed above, intravenous fluid therapy might significantly alter the acid–base and electrolyte equilibrium of both blood and CSF [47–51]. Finally, a potential source of variability among CSF samples could derive from the collection timing. The CSF samples of the SAH population were collected as soon as clinically feasible, i.e., reliant on the clinical decision to place an external ventricular drain.

## Conclusions

Patients with SAH have a localized CSF acidosis, characterized by a reduced CSF SID and mainly caused by increased lactate concentrations. CSF PCO_2_ is reduced to restore a normal CSF pH. This compensatory mechanism might explain the high incidence of arterial HA in patients with spontaneous SAH.

## Supplementary Information

Below is the link to the electronic supplementary material.

Supplementary file1 (DOCX 189 KB)
