# Supplementary material for: Cerebrospinal Fluid and Arterial Acid–Base Equilibrium of Spontaneously Breathing Patients with Aneurismal Subarachnoid Hemorrhage
Source: Neurocrit Care. 2022 Feb 23;37(1):102–10. doi: 10.1007/s12028-022-01450-1 (PMC9283163; doi:10.1007/s12028-022-01450-1)
Supplement: Supplementary file 1 — Supplementary file1 (DOCX 189 KB) [file 12028_2022_1450_MOESM1_ESM.docx]

**Cerebrospinal fluid and arterial acid-base equilibrium of spontaneously breathing patients with aneurismal subarachnoid hemorrhage**

Thomas Langer^1,2^, MD; Francesco Zadek^1,3^, MD; Marco Carbonara^4^, MD;
Alessio Caccioppola^3,4^, MD; Serena Brusatori^3^, MD; Tommaso Zoerle^3,4^, MD;
Francesco Bottazzini^4^, MD; Chiara Ferraris Fusarini^5^, BSc; Adriana di Modugno^5^,
Alberto Zanella^3,4^, MD; Elisa R. Zanier^6^, MD; Roberto Fumagalli^1,2^, MD;
Antonio Pesenti^3,4^, MD; Nino Stocchetti^3,4^, MD.

^1^ Department of Medicine and Surgery, University of Milan-Bicocca, Monza, Italy

^2^ Department of Anesthesia and Intensive Care Medicine, Niguarda Ca' Granda, Milan, Italy

^3^ Department of Pathophysiology and Transplantation, University of Milan, Milan, Italy

^4^ Department of Anesthesia, Critical Care and Emergency, Fondazione IRCCS Ca' Granda Ospedale Maggiore Policlinico, Milan, Italy

^5^ Central Laboratory, Fondazione IRCCS Ca' Granda, Ospedale Maggiore Policlinico

^6^ Laboratory of Acute Brain Injury and Therapeutic Strategies, Department of Neuroscience, Istituto Di Ricerche Farmacologiche Mario Negri IRCCS

***Online Supplementary Results***

**Table S1. Comorbidities of the enrolled patients.**

| **Variables** | **Controls  n = 25** | **SAH n = 20** | **p value** |
| --- | --- | --- | --- |
| **Systemic Hypertension ̶ n (%)** | 7 (28) | 9 (45) | 0.09 |
| **Active Smoker ̶ n (%)** | 3 (12) | 10 (50) |  |
| **Psychiatric Disease ̶ n (%)** | 2 (8) | 0 (0) |  |
| **Diabetes ̶ n (%)** | 1 (4) | 0 (0) |  |
| **Chronic obstructive Pulmonary disease ̶ n (%)** | 0 (0) | 0 (0) |  |
| **Chronic kidney disease ̶ n (%)** | 0 (0) | 0 (0) |  |

Chronic obstructive pulmonary disease and chronic kidney disease were reported to underline the absence of these comorbidities.

**Table S2: Baseline characteristics of the SAH population.**

| **Variables** | **Non-Hypocapnic Alkalosis n = 12** | **Hypocapnic Alkalosis  n = 8** | **P value** |
| --- | --- | --- | --- |
| **Age ̶ years** | 59 ± 10 | 54 ± 4 | 0.34 |
| **Female sex - no. (%)** | 10 (83) | 6 (75) | 0.65 |
| **Body mass index ̶ kg/m^2^** | 24 [23, 29] | 25 [23, 28] | 0.41 |
| **GCS** | 14 [8.5, 15] | 11 [8, 14.5] | 0.38 |
| **WFNS scale** | 3.5 [1, 4] | 3.5 [2, 4] | 0.75 |
| **Modified FISHER scale** | 4 [1, 4] | 4 [3, 4] | 0.58 |
| **Vasospasm ̶ no. (%)** | 3 (25) | 3 (38) | 0.45 |
| **Sample Day** | 2 [1.5, 3] | 2 [2, 5] | 0.32 |
|  |  |  |  |
| **Ventilation ̶ no. (%)** |  |  |  |
| **Spontaneuos breathing** | 7 (58) | 3 (38) | 0.36 |
| **Assisted ventilation** | 5 (42) | 5 (63) |  |
|  |  |  |  |
| **Clinical outcomes** |  |  |  |
| **GOS-E** | 7 [3, 8] | 6 [1, 6] | 0.31 |

Comparisons of main baseline characteristics of SAH patient divided on the presence of hypocapnic alkalosis. Data are presented ad mean ± standard deviation, median [interquartile range] or frequency (percentage).
*Definition of abbreviations:* GOS-E: Glasgow Outcome Scale Extended; GCS**:** Glasgow Coma Scale; SAH: aneurismal subarachnoid hemorrhage; WFNS: World Federation of Neurosurgical Societies scale.

**Table S3: Acid-base characteristics of plasma of the SAH population.**

| **Variables** | **Non-Hypocapnic Alkalosis n = 12** | **Hypocapnic Alkalosis  n = 8** | **p-value** |
| --- | --- | --- | --- |
| **pH** | 7.43 ± 0.04 | 7.48 ± 0.03 | 0.004 |
| **PCO_2_ ̶ mm Hg** | 40 ± 5 | 32 ± 2 | <0.001 |
| **A_TOT_ ̶ mmol/L** | 13.8 ± 1.5 | 13.1 ± 1.6 | 0.32 |
| **SID ̶ mmol/L** | 38.6 ± 4.1 | 35.1 ± 1.7 | 0.04 |
| **pO_2_ ̶ mm Hg** | 107 ± 22 | 116 ± 35 | 0.48 |
| **Na^+^ ̶ mmol/L** | 142 ± 4 | 142 ± 3 | 0.83 |
| **K^+^ ̶ mmol/L** | 3.6 ± 0.2 | 3.4 ± 0.2 | 0.14 |
| **Ca^2+^ ̶ mmol/L** | 1.19 ± 0.04 | 1.15 ± 0.02 | 0.04 |
| **Mg^2+^ ̶ mmol/L** | 0.84 ± 0.08 | 0.86 ± 0.07 | 0.53 |
| **Cl^-^ ̶ mmol/L** | 110 ± 6 | 114 ± 4 | 0.13 |
| **Lac^-^ ̶ mmol/L** | 1.0 ± 0.6 | 1.0 ± 0.4 | 0.89 |
| **HCO_3_^-^  ̶ mmol/L** | 26.5 ± 2.9 | 24.0 ± 2.3 | 0.06 |
| **Phosph^-^ ̶ mEq/L** | 1.49 ± 0.52 | 1.49 ± 0.49 | 0.99 |
| **Alb^-^ ̶ mEq/L** | 9.7 ± 1.1 | 9.3 ± 1.0 | 0.47 |
| **SBE ̶ mmol/L** | 2.5 ± 2.9 | 0.4 ± 1.3 | 0.08 |
| **Osmolarity ̶ mOsm/L** | 290 ± 9 | 292 ± 5 | 0.70 |
| **Gluc ̶ mg/dL** | 128 ± 22 | 131 ± 17 | 0.79 |
| **Hb ̶ g/dL** | 11.8 ± 1.7 | 10.8 ± 1.11 | 0.17 |
| **RBC ̶ cells*10^6^/µL** | 3.85 ± 0.83 | 3.38 ± 0.42 | 0.16 |
| **WBC ̶ cells/µL** | 9872 ± 1707 | 10716 ± 3229 | 0.45 |

Data are expressed as mean ± standard deviation.
*Definition of abbreviations:* Alb^-^ = ionized albumin concentration; A_TOT_ = total amount of weak, non-carbonic acids; Ca^2+^= ionized calcium concentration; Cl^-^=chloride concentration; Gluc = glucose concentration; Hb = Hemoglobin concentration; HCO_3_^-^ = bicarbonate concentration; K^+^= potassium concentration; Mg^2+^ = magnesium concentration; Na^+^= sodium concentration; PCO_2_= partial pressure of carbon dioxide; Phosph^-^ = ionized phosphate concentration; PO_2_= partial pressure of oxygen; RBC = Red Blood Cell count; SBE = Standard base Excess; SID = Strong Ion Difference; WBC = White Blood Cell count.

**Table S4. Multilinear regression model.**

| CSF pH | β | | SE | t-value | | **P value** | 95% CI | | | |
| --- | --- | --- | --- | --- | --- | --- | --- | --- | --- | --- |
| CSF SID | 0.017 | | 0.003 | 6.01 | | <0.001 | [0.011, 0.023] | | | |
| CSF PCO_2_ | -0.011 | | 0.002 | -6.35 | | <0.001 | [-0.014, -0.007] | | | |
| Constant | 7.384 | | 0.084 | 88.04 | | <0.001 | [7.207, 7.561] | | | |
|  |  | |  |  | |  |  | |  | |
| r^2^ | | 0.791 | | | Number of patients | | | 20 | |  |
| F-test | | 32.215 | | | Prob > F | | | <0.001 | |  |

Multilinear regression model. Dependent variable CSF pH, independent variable CSF SID, CSF PCO_2._ β coefficient,
*Definition of abbreviations:* CI: confidence interval; CSF = cerebrospinal fluid; SE: standard error; SID = Strong Ion Difference; PCO_2_= partial pressure of carbon dioxide.

**Table S5. Multilinear regression model.**

| CSF pH | β | | SE | t-value | | **P value** | 95% CI |
| --- | --- | --- | --- | --- | --- | --- | --- |
| CSF Na^+^ | 0.003 | | 0.006 | 0.53 | | 0.604 | [-0.009, 0.015] |
| CSF Cl^-^ | -0.008 | | 0.005 | -1.75 | | 0.099 | [-0.018, 0.002] |
| CSF Lactate | -0.02 | | 0.008 | -2.45 | | 0.026 | [-0.037, -0.003] |
| Constant | 7.952 | | 0.442 | 17.98 | | <0.001 | [7.014, 8.89] |
|  | |  | | |  | |  |
| r^2^ | | 0.554 | | | Number of patients | | 20 |
| F-test | | 6.631 | | | Prob > F | | 0.004 |

Multilinear regression model. Dependent variable CSF pH, independent variable CSF Na, CSF Cl, CSF Lactate_._ β coefficient,
*Definition of abbreviations:* CI: confidence interval; Cl^-^: chloride concentration; CSF: cerebrospinal fluid, Na^+^: sodium concentration; SE: standard error.

**Table S6. CSF Variables divided by the development of clinical vasospasm.**

| **Variables** | **No vasospasm  n = 14** | **Vasospasm n = 6** | **p value** |
| --- | --- | --- | --- |
| **pH** | 7.36 ± 0.1 | 7.33 ± 0.1 | 0.21 |
| **PCO_2_ ̶ mm Hg** | 40 ± 4 | 41 ± 4 | 0.63 |
| **A_TOT_ ̶ mmol/L** | 1.2 ± 0.4 | 1.4 ± 0.5 | 0.39 |
| **SID ̶ mmol/L** | 23.2 ± 1.7 | 22.6 ± 3.4 | 0.61 |
| **Na^+^ ̶ mmol/L** | 143 ± 4 | 144 ± 3 | 0.46 |
| **K^+^ ̶ mmol/L** | 2.5 ± 0.4 | 2.7 ± 0.8 | 0.39 |
| **Ca^2+^ ̶ mmol/L** | 1.0 ± 0.0 | 1.0 ± 0.1 | 0.52 |
| **Mg^2+^ ̶ mmol/L** | 2.3 ± 0.1 | 2.3 ± 0.1 | 0.55 |
| **Cl^-^ ̶ mmol/L** | 123 ± 5 | 125 ± 4 | 0.48 |
| **Lac^-^ ̶ mmol/L** | 3.0 ± 1.3 | 3.7 ± 1.3 | 0.36 |
| **HCO_3_^-^  ̶ mmol/L** | 22.1 ± 2.0 | 21.0 ± 3.2 | 0.36 |
| **Phosph^-^ ̶ mEq/L** | 0.7 ± 0.2 | 0.7 ± 0.2 | 0.90 |
| **Alb^-^ ̶ mEq/L** | 0.3 ± 0.1 | 0.4 ± 0.4 | 0.26 |
| **Osmolarity ̶ mOsm/L** | 289 ± 8 | 291 ± 5 | 0.62 |
| **Hb ̶ g/dL** | 0.3 ± 0.3 | 0.4 ± 0.2 | 0.44 |
| **RBC ̶ cells*10^6^/µL** | 0.1 ± 0.1 | 0.1 ± 0.1 | 0.90 |
| **WBC ̶ cells/µL** | 0.3 ± 0.5 | 0.5 ± 0.7 | 0.56 |
| **Delta SID ^-^  ̶ mmol/L** | 14.6 ± 3.0 | 13.3 ± 2.9 | 0.39 |
| **Delta PCO_2_ ̶ mm Hg** | 3.1 ± 4.6 | 4.1 ± 6.9 | 0.69 |

Data are expressed as mean ± standard deviation.
*Definition of abbreviations:* Alb^-^ = ionized albumin concentration; A_TOT_ = total amount of weak, non-carbonic acids; Ca^2+^= ionized calcium concentration; Cl^-^=chloride concentration; CSF = cerebrospinal fluid; Delta PCO_2_ = CSF-to-plasma partial pressure of carbon dioxide; Delta SID = CSF-to-plasma Strong Ion Difference; Hb = Hemoglobin concentration; HCO_3_^-^ = bicarbonate concentration; K^+^= potassium concentration; Lac^-^ = Lactate concentration; Mg^2+^ = magnesium concentration; Na^+^= sodium concentration; PCO_2_= partial pressure of carbon dioxide; Phosph^-^ = ionized phosphate concentration; RBC = Red Blood Cell count; SID = Strong Ion Difference; WBC = White Blood Cell count.

**Table S7 Plasma Variables divided by the development of clinical vasospasm**

| **Variables** | **No vasospasm  n = 14** | **Vasospasm n = 6** | **p value** |
| --- | --- | --- | --- |
| **pH** | 7.46 ± 0.03 | 7.43 ± 0.06 | 0.20 |
| **PCO_2_ ̶ mm Hg** | 37 ± 5 | 37 ± 8 | 0.97 |
| **A_TOT_ ̶ mmol/L** | 13.6 ± 1.6 | 13.3 ± 1.7 | 0.75 |
| **SID ̶ mmol/L** | 37.8 ± 3.7 | 35.9 ± 3.7 | 0.32 |
| **PO_2_ ̶ mm Hg** | 106 ± 26 | 123 ± 30 | 0.20 |
| **Na^+^ ̶ mmol/L** | 141 ± 4 | 144 ± 3 | 0.22 |
| **K^+^ ̶ mmol/L** | 3.5 ± 0.2 | 3.4 ± 0.1 | 0.29 |
| **Ca^2+^ ̶ mmol/L** | 1.2 ± 0.0 | 1.2 ± 0.0 | 0.57 |
| **Mg^2+^ ̶ mmol/L** | 1.7 ± 0.1 | 1.7 ± 0.2 | 0.36 |
| **Cl^-^ ̶ mmol/L** | 110 ± 6 | 114 ± 4 | 0.13 |
| **Lac^-^ ̶ mmol/L** | 0.9 ± 0.4 | 1.1 ± 0.7 | 0.52 |
| **HCO_3_^-^  ̶ mmol/L** | 26.2 ± 2.9 | 23.9 ± 2.5 | 0.12 |
| **Phosph^-^ ̶ mEq/L** | 1.5 ± 0.5 | 1.5 ± 0.5 | 0.73 |
| **Alb^-^ ̶ mEq/L** | 9.7 ± 1.2 | 9.3 ± 0.9 | 0.43 |
| **SBE ̶ mmol/L** | 2.3 ± 2.6 | 0.1 ± 1.9 | 0.09 |
| **Osmolarity ̶ mOsm/L** | 290 ± 8 | 293 ± 6 | 0.37 |
| **Hb ̶ g/dL** | 11.3 ± 1.8 | 11.6 ± 1.0 | 0.71 |
| **RBC ̶ cells*10^6^/µL** | 3.7 ± 0.8 | 3.5 ± 0.6 | 0.45 |
| **WBC ̶ 10^3^cells/µL** | 9.8 ± 2.1 | 11.1 ± 3.0 | 0.27 |

Data are expressed as mean ± standard deviation.
*Definition of abbreviations:* Alb^-^ = ionized albumin concentration; A_TOT_ = total amount of weak, non-carbonic acids; Ca^2+^= ionized calcium concentration; Cl^-^=chloride concentration; Hb = Hemoglobin concentration; HCO_3_^-^ = bicarbonate concentration; K^+^= potassium concentration; Lac^-^ = Lactate concentration; Mg^2+^ = magnesium concentration; Na^+^= sodium concentration; PCO_2_= partial pressure of carbon dioxide; Phosph^-^ = ionized phosphate concentration; PO_2_= partial pressure of oxygen; RBC = Red Blood Cell count; SBE = Standard base Excess; SID = Strong Ion Difference; WBC = White Blood Cell count.

**Figure S1. Scatter plot graph of CSF SID and arterial pH in SAH patients.**


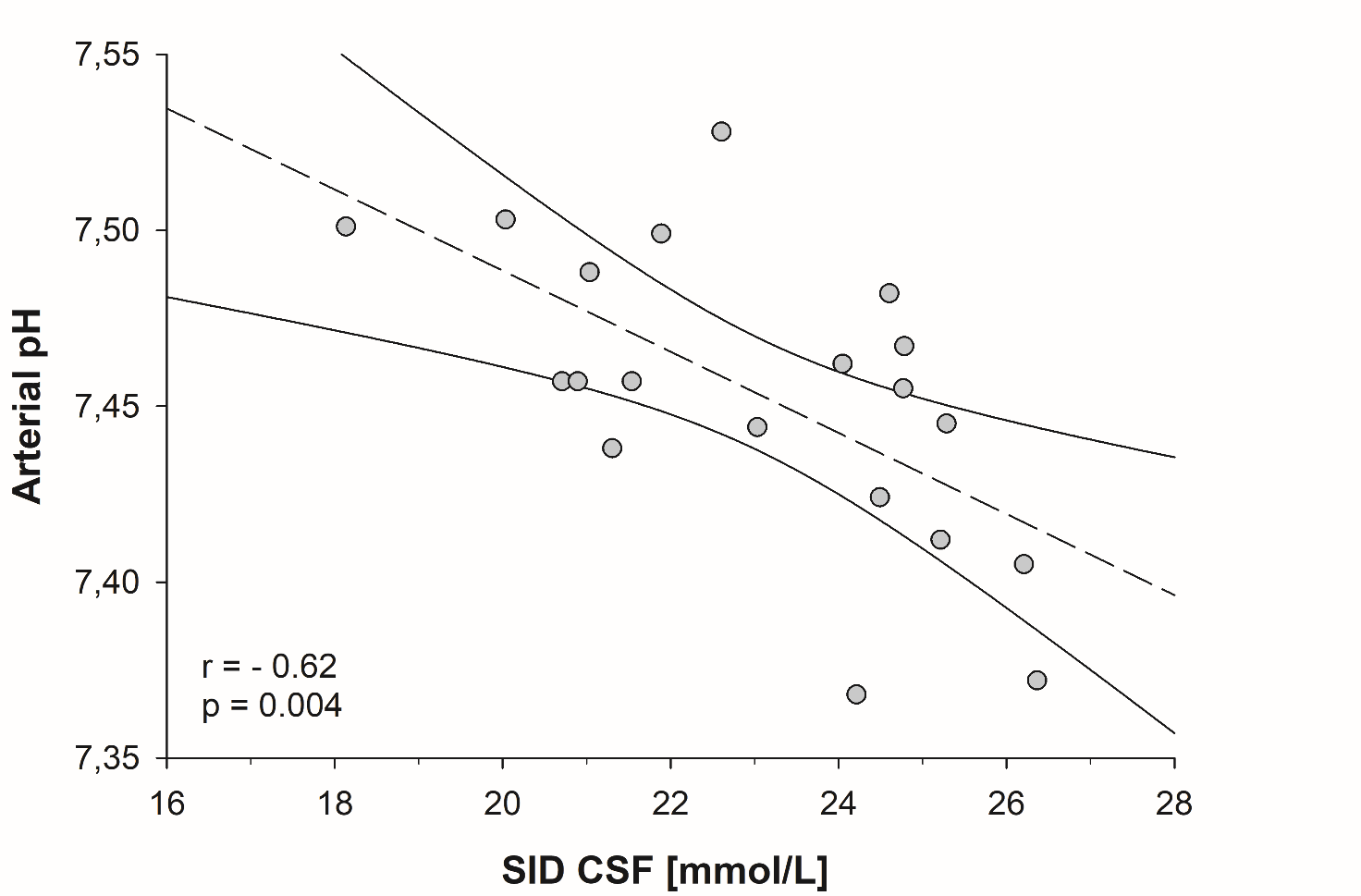


Scatter plot representing the association between CSF SID and arterial pH in SAH patients. Linear regression model (dashed line) is represented with 95% confidence interval. Equation of the linear model: Arterial pH = -0.01* CSF SID + 7.7.

*Definition of abbreviations:* CSF = cerebrospinal fluid; SAH: aneurismal subarachnoid hemorrhage; SID = Strong Ion Difference.
